# The impact of caregiver burden on quality of life in family caregivers of patients with advanced cancer: a moderated mediation analysis of the role of psychological distress and family resilience

**DOI:** 10.1186/s12889-024-18321-3

**Published:** 2024-03-15

**Authors:** Panpan Cui, Ming Yang, Hengyu Hu, Chunyan Cheng, Xinyi Chen, Jiaoxia Shi, Shifeng Li, Changying Chen, Hongmei Zhang

**Affiliations:** 1 Department of Nursing, Henan Provincial Key Medicine Laboratory of Nursing, Henan Provincial People’s Hospital, Zhengzhou University People’s Hospital, No. 7 Weiwu Road, Zhengzhou, China; 2 School of Nursing, Zhengzhou University, Zhengzhou, China; 3 Nursing Department, Xinyang Central Hospital, Xinyang, China; 4 Hemangiomatology Department, Henan Provincial People’s Hospital, Zhengzhou University People’s Hospital, Zhengzhou, China; 5 Medical Oncology, Henan Provincial People’s Hospital, Zhengzhou University People’s Hospital, Zhengzhou, China; 6 Medical Oncology, People’s Hospital of Jiaozuo City, Jiaozuo, China; 7 Medical Oncology, Xinyang Central Hospital, Xinyang, China; 8 The First Affiliated Hospital of Zhengzhou University, No. 1 Jianshe Dong Road, Zhengzhou, China; 9 Institute for Hospital Management of Henan Province, Zhengzhou, China

**Keywords:** Caregiver burden, Family resilience, Psychological distress, Quality of life, Advanced cancer, Moderated mediation analysis

## Abstract

**Background:**

The caregiver burden frequently experienced by family members tending to advanced cancer patients significantly impacts their psychological well-being and quality of life (QoL). Although family resilience might function as a mitigating factor in this relationship, its specific role remains to be elucidated. This study aims to probe the mediating effect of psychological distress on the relationship between caregiver burden and QoL, as well as the moderating effect of family resilience.

**Methods:**

A cross-sectional study was conducted between June 2020 and March 2021 in five tertiary hospitals in China. Data were collected on caregiver burden, family resilience, psychological distress (including anxiety and depression), and QoL. Moderated mediation analysis was performed.

**Results:**

Data analysis included 290 caregivers. It confirmed the mediating role of psychological distress in the caregiver burden-QoL relationship (*P* < 0.001). Both overall family resilience and the specific dimension of family communication and problem-solving (FCPS) demonstrated significant moderating effects on the “psychological distress/anxiety—QoL” paths (*P* < 0.05). The utilization of social and economic resources (USER) significantly moderated the association between depression and QoL (*P* < 0.05).

**Conclusions:**

The study corroborates psychological distress's mediation between caregiver burden and QoL and family resilience's moderation between psychological distress and QoL. It underscores the need for minimizing psychological distress and bolstering family resilience among caregivers of advanced cancer patients. Accordingly, interventions should be tailored, inclusive of psychological assistance and promotion of family resilience, particularly focusing on FCPS and USER, to augment the caregivers' well-being and QoL.

**Supplementary Information:**

The online version contains supplementary material available at 10.1186/s12889-024-18321-3.

## Introduction

Caregivers of advanced cancer patients, due to the complex and chronic nature of the illness, shoulder significant responsibilities such as daily care, medication management, pain control, and emotional support [1, 2]. These demanding tasks often result in physical exhaustion and psychological burden, leading to considerable psychological distress [3, 4]. This distress, primarily manifested as anxiety and depression, is exacerbated by the unpredictable progression of the disease and the associated pain of the patient, yielding significant repercussions on the caregivers' quality of life (QoL) [5]. Past research has indeed confirmed the prevalence of such distress among caregivers of advanced cancer patients [6].

This psychological distress can considerably impinge on caregivers' QoL, affecting daily functioning and potentially instigating adverse impacts on family relationships, social interactions, and work productivity [5]. This can precipitate a deterioration in overall QoL and induce negative psychological and physiological responses. Additionally, psychological distress may mediate the relationship between the caregiving burden and QoL, depleting the caregivers' psychological resources and impacting their ability to cope, thereby intensifying the negative impact of the burden and diminishing their QoL. Despite this, most previous research have primarily focused on the direct relationships between caregiving burden, psychological distress, and QoL, providing limited insights into the mechanisms through which caregiving burden influences QoL [4, 5, 7, 8]. To rectify this, a comprehensive exploration of the mediating role of psychological distress between caregiving burden and QoL is necessary. Such an investigation could furnish practical guidance for targeted interventions aimed at alleviating psychological distress, enhancing caregivers' coping abilities, and ultimately mitigating the negative influence of caregiving burden on their QoL. Moreover, enhancing caregivers' psychological well-being could improve the efficacy and quality of caregiving services, ensuring more attentive and comprehensive support for patients [9].

Notably, not all caregivers succumb to feelings of helplessness or struggle to cope with the burden and psychological distress. Some demonstrate significant family resilience, which can positively affect their experiences and QoL during caregiving in the context of advanced cancer [10]. Family resilience refers to the ability of a family to withstand and recover from adversity [11, 12]. Emphasizing the family belief system (i.e., meaning-making, positive outlook, transcendence), communication processes (i.e., clear information, emotional sharing, collaborative problem solving), and organizational processes (i.e., flexibility, connectedness and mutual support, social and economic resources), Walsh's theory of family resilience offers an interpretive lens [13]. From a positive psychology perspective, family resilience elucidates how families perceive and respond to stress and adversity, their support networks, and their positive coping strategies; stronger family resilience often results in effective resource utilization, emotional stability, reduced psychological distress, and an enhanced QoL [13, 14]. This fosters a supportive environment for families dealing with the challenges of advanced cancer. Past research suggests a negative association between higher family resilience and caregiving burden [15], and a positive association with caregivers' QoL [16]. Although family resilience may potentially buffer the relationship between caregiving burden and QoL, the specific nature of this association requires further investigation.

This study addresses this gap, examining the mediating role of psychological distress between caregiving burden and QoL and the moderating effect of family resilience in this mediation process. To assess family resilience, the Family Resilience Assessment Scale (FRAS) will be employed, a comprehensive tool developed by Sixbey [17] that encompasses six domains, each reflecting different aspects of family resilience. Through this investigation, the specific domain(s) influencing the mediation process will be identified. Gaining a better understanding of these mediating and moderating mechanisms will facilitate the development of targeted and personalized support for caregivers, ultimately enhancing their QoL and overall caregiving experiences.

### Theoretical framework

**Fig. 1:**
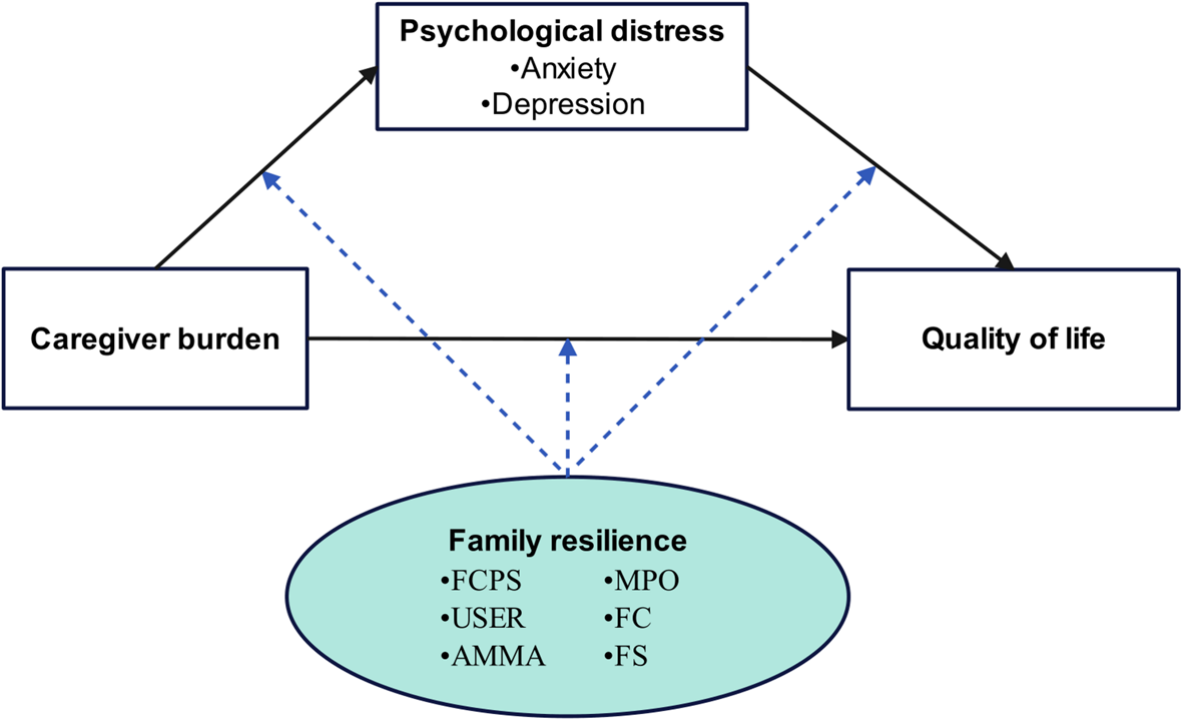
The hypothetical moderated mediation model. FCPS: Family communication and problem solving; USER: Utilization of social and economic resources; AMMA: Ability to make meaning of adversity; MPO: Maintaining a positive outlook; FC: Family connectedness; FS: Family spirituality

The proposed moderated mediation model, as depicted in Fig. 1, posits a negative correlation between caregiver burden and QoL. The underlying framework for this mediation model is based on Lazarus’ cognitive appraisal theory of stress [18]. According to this theory, increased caregiver burden is expected to predict higher levels of psychological distress, leading to poorer QoL. In line with the cognitive appraisal theory of stress, family resilience is closely related to the process of secondary appraisal and coping [13, 19]. Thus, family resilience is expected to moderate this mediation model. Specifically, by strengthening the family belief system, caregivers may develop constructive beliefs and positive attitudes towards cancer. This, in turn, enables them to effectively recognize and utilize inherent and potential strengths and resources within their family network to navigate the challenges posed by cancer.

Based on the theoretical framework, this study aims to elucidate the associations and underlying mechanisms linking caregiver burden and the QoL among caregivers of advanced cancer patients. Specifically, it focuses on the potential mediating role of psychological distress, along with its sub-dimensions (depression and anxiety), and also examine the moderating impact of perceived family resilience, including its sub-dimensions, on these associations. Two hypotheses are proposed:

- H1: Psychological distress and its sub-factors (depression and anxiety) mediate the relationship between caregiver burden and QoL.
- H2: Family resilience and its sub-dimensions moderate the mediation models of “caregiver burden—psychological distress/depression/anxiety—QoL”, either in the first path, second path, and/or direct path.

## Materials and methods

### Design and settings

A multi-center, cross-sectional study was conducted in the oncology wards of five tertiary hospitals in Henan Province, China, between June 2020 and March 2021.

### Participants and procedure

Caregivers of advanced cancer patients were included if they: 1) cared for advanced cancer patients aged over 18, diagnosed with stage IV cancer, including prevalent solid tumors like lung cancer, esophageal cancer, breast cancer, and colorectal cancer, as well as hematologic malignancies like lymphoma, 2) were aged over 18 themselves, 3) were identified by patients as the primary, unpaid caregiver, and 4) provided informed consent. These criteria were established to uphold legal and ethical considerations and specifically target individuals playing a substantial caregiving role. Caregivers with severe physical or mental illnesses, as well as caregivers of patients with unusual cancers such as skin melanoma, were excluded. Aiming for a sample size of over 200, as suggested for structural equation modeling [20], 330 caregivers were invited to participate, with a final sample of 290 caregivers.

Caregivers were recruited using convenience and purposive sampling techniques by five trained research assistants (registered nurses), one from each hospital. When participants required assistance with the questionnaire, the research assistants provided support by reading the items aloud and objectively recording the responses. The research assistants meticulously extracted clinical data related to the patients from the medical record system. To bolster participant engagement and ensure the quality of responses, research assistants strategically distributed the questionnaires in the afternoon, a period with reduced treatment activities. Furthermore, the research assistants remained readily available to clarify any perplexing items, ensuring that participants fully comprehended the questionnaire content before proceeding. Following completion, the research assistants scrupulously reviewed the questionnaires to identify any missing items and promptly requested participants to provide responses for any omissions. Questionnaires with more than 10% missing items or displaying patterned responses were systematically excluded from the analysis to uphold data integrity. Ethical approval was granted by the ethics committee of the corresponding university. All caregivers provided informed consent prior to questionnaire completion.

### Measures

A self-administered questionnaire gathered information on caregivers' socio-demographic characteristics, caregiving burden, psychological distress, QoL, and family resilience. Patients' age, sex, and clinical data (i.e., primary cancer type and time since advanced cancer diagnosis), were extracted from the medical system by research assistants.

#### QoL

The 8-item SF-8 (the short form 8 health survey) was adopted to assess caregivers’ QoL [21]. Caregivers were asked, i.e., “Overall, your health status in the past 4 weeks is”. Each item uses a Likert 5- or 6- level scoring method and measures one health dimension. The raw scores of each dimension are converted into T-scores (mean = 50, standard deviation = 10) that range from 0 to 100. SF-8 has two summary scores, that is, the physical component summary (PCS) and the mental component summary (MCS) [22]. The total scores are calculated as the average means of all dimensions, with higher scores indicating better health. The Cronbach’s alpha was 0.871 in this study.

#### Caregiver burden

Caregiver burden was assessed using the 22-item Zarit Burden Interview (ZBI) [23]. ZBI consists of two dimensions, that is, personal burden (e.g., Do you feel your relative asks for more help than he/she needs?) and responsibility burden (e.g., The patient has affected the relationship between you and your family and friends). Responses ranged from 0 (almost none) to 4 (always), yielding a total score of 0 ~ 88. Higher scores represent worse burden. The Cronbach’s alpha was 0.910 in this study.

#### Psychological distress

The 4-item patient health questionnaire (PHQ-4) was used to measure caregivers’ psychological distress [24]. There are two 2-item subscales, measuring depression or anxiety, respectively. Participants were asked, “Over the last 2 weeks, how often have you been bothered by the following problems?” Responses range from from 0-”not at all” to 3-”nearly everyday”. The total scores for depression and anxiety range from 0 to 6 and the total scores for PHQ-4 range from 0 to 12. The Cronbach’s alpha was 0.862 in this study.

#### Family resilience

51-item Chinese version of Family Resilience Assessment Scale (FRAS) was adopted to measure perceived family resilience of caregivers [17, 25]. FRAS consists of six subscales, including (1) family communication and problem solving (FCPS) (e.g., ‘Our family structure can flexibly cope with unexpected events’), (2) utilization of social and economic resources (USER) (e.g., ‘We can rely on people in the community’), (3) ability to make meaning of adversity (AMMA) (e.g., ‘We accept stress events as part of our lives’), (4) maintaining a positive outlook (MPO) (e.g., ‘We believe that we can deal with our problems’), (5) family connectedness (FC) (e.g., ‘We express love and affection to family members’), and (6) family spirituality (FS) (e.g., ‘We participate in religious services’). Responses range from 1- ‘strongly disagree’ to 4- ‘strongly agree’. Four items (30, 34, 42, 47) are reverse scored before summing all items to yield a total score. The content validity index for the Chinese version FRAS is 0.97, and the Cronbach’s alpha is 0.944. The Cronbach’s alpha coefficients for each dimension range from 0.700 to 0.951, with a split-half reliability of 0.807 and a test–retest reliability of 0.917 in Chinese cancer families [25]. The total scores range from 51 ~ 204, with higher scores indicating higher levels of family resilience. The Cronbach’s alpha was 0.936 in this study.

#### Social-demographic characteristics

Several sociodemographic characteristics were gathered, including caregivers’ age, sex, marital status, education level, employment status, place of residence, monthly household income per capita, presence of chronic diseases, relationship with patients, previous caregiving experience, type of caregiving (sole caregiver or part of a caregiving team), length of caregiving, and daily caregiving hours. These variables, which might significantly predict caregivers' QoL, were employed as control variables in the models.

### Statistical analysis

The proposed model was examined using SPSS version 21.0, with Hayes's PROCESS macro version 3.4 employed for bootstrapping indirect effects [26]. A regression model of QoL was performed and the sociodemographic characteristics which could statistically significantly predicted QoL would be adpoted as control variables in the mediation and moderation models.

To test hypothesis 1, several structural equation models were employed using Model 4 of PROCESS macro. In the mediation models, each yielded two equations: the first examining psychological distress or its sub-factors (depression / anxiety) as the dependent variable, with caregiver burden as the independent variable, and the second with QoL as the dependent variable and all other variables (predictors and control variables) as independent. Subsequently, the direct and indirect effects of the predictor on QoL were calculated. A direct effect is represented by the regression coefficient (beta) of the predictor (caregiver burden in this study) on a dependent variable (QoL). Conversely, an indirect effect is computed as the product of two path coefficients. The mediation effect is classified as ‘complete mediation’ when the observed indirect effect is statistically significant while the direct effect is not statistically significant. On the other hand, ‘partial mediation’ is indicated when both the indirect and direct effects are statistically significant [26].

Hypothesis 2 was tested by initially estimating the moderating effect of family resilience (FR) in the mediation model (caregiver burden [CGB]-psychological distress [PD]—QoL) using Model 59 of PROCESS macro. Statistically significant effects of both interaction terms (CGB * FR, PD * FR) indicated the presence of moderation effects. Where only CGB * FR or PD * FR was statistically significant, Model 7 or Model 14 was conducted respectively, to further probe the moderating effect. The moderating effects were also explored using simple slope tests. The relationships between caregiver burden and psychological distress, as well as psychological distress and QoL, were plotted at varying levels of the moderator (family resilience). Further exploration of the moderating effects of family resilience on different sub-factors of the mediator (depression and anxiety) was conducted separately for each dimension of family resilience.

Finally, the proposed moderated-mediation models were tested using bias-corrected bootstrap method to explore how varying levels of family resilience and its sub-dimensions affect the caregiver burden- psychological distress/depression/anxiety- QoL relationship. Specifically, one standard deviation above and one standard deviation below the mean of family resilience, representing high and low levels, respectively, were examined. Bootstrapping with 5000 samples was used to correct the 95% confidence interval (CI) of the effect. The presence of a moderating effect was confirmed if the 95% CI of the effect size did not include zero.

## Results

### Characteristics of participants

Of the 300 questionnaire returned (90.9%), 290 (87.9%) were analyzed. The sample characteristics are presented in Table 1. Patients' average age was 55.4 years (SD 15.3), and caregivers' average age was 44.6 years (SD 13.5). Of the caregivers, 52.1% were female, and the majority were married, with adult children accounting for 43.8%. Approximately 43.8% of caregivers were employed. Additionally, 49.7% reported a monthly household income per capita greater than 3000 RMB, and 50.7% were from rural areas. Among the patients, 76.2% were diagnosed with a solid tumor as their primary cancer, and the median time since advanced cancer diagnosis was eight months. Almost half of the caregivers had been providing care for patients for at least six months. Detailed scores of caregivers on different variables are shown in Table 2.

**Table 1:** Sample characteristics (*N* = 290)

**Variables**		Caregiver		Patient	
		**Frequency**	**Percentage**	**Frequency**	**Percentage**
**Age (year)**	M ± SD	44.6 ± 13.5		55.4 ± 15.3	
	< 45	151	52.1	64	22.1
	45 ~ 59	99	34.1	102	35.2
	≥ 60	40	13.8	124	42.7
**Sex**	Male	139	47.9	159	54.8
	Female	151	52.1	131	45.2
**Primary cancer (patient)**	Solid tumor			221	76.2
	Hematologic tumor			69	23.8
**Time since advanced cancer diagnosis/month (patient)**	< 8 months^a^			137	47.2
	≥ 8 months			153	52.8
**Marital status**	Married	255	87.9		
	Unmarried/divorced/widowed	35	12.1		
**Education Level**	≤ Middle school	128	44.1		
	> Middle school	162	55.9		
**Working status**	Employed	127	43.8		
	Unemployed/retired	163	56.2		
**Place of residence**	Rural	147	50.7		
	Urban	143	49.3		
**The presence of chronic diseases**	Yes	52	17.9		
	No	238	82.1		
**Monthly household income per capita (RMB)**	< 3000	146	50.3		
	≥ 3000	144	49.7		
**Caregiver type**	Spouse caregiver	121	41.7		
	Non-spouse caregiver	169	58.3		
	Adult children	127	43.8		
	Others (parents/siblings/ grandchildren)	42	14.5		
**Whether they had similar caregiving experience**	Yes	68	23.4		
	No	222	76.6		
**Type of caregiving**	Care for patients alone	159	54.8		
	Care for patients with others	131	45.2		
**Length of care (month)**	< 6	158	54.4		
	6 ~	66	22.8		
	≥ 12	66	22.8		
**Caregiving hours per day (hour)**	< 6	96	33.1		
	7 ~	79	27.2		
	12 ~	37	12.8		
	18 ~ 24	78	26.9		

*M* Mean, *SD* Standard deviation

^a^The median time since advanced cancer diagnosis was 8 months

**Table 2:** Caregivers' scores on various variables (*N* = 290)

Variables	M ± SD
***Outcome variable***	
QoL (SF-8)	79.08 ± 15.32
***Predictor variable***	
Caregiver burden (ZBI)	31.16 ± 14.32
***Mediator variable***	
Psychological distress (PHQ-4)	2.91 ± 2.63
Depression	1.37 ± 1.35
Anxiety	1.54 ± 1.48
***Moderator variable***	
Family resilience (FRAS)	150.44 ± 13.95
FCPS	74.63 ± 8.02
USER	23.24 ± 2.92
AMMA	9.28 ± 1.41
MPO	18.40 ± 2.24
FC	16.02 ± 1.97
FS	8.87 ± 2.67

*M* Mean, *SD* Standard deviation, *QoL* Quality of life, *FCPS* Family communication and problem solving, *USER* Utilization of social and economic resources, *AMMA* Ability to make meaning of adversity, *MPO* Maintaining a positive outlook, *FC* Family connectedness, *FS* Family spirituality, *SF-8* the short form 8 health survey, *ZBI* Zarit Burden Interview, *PHQ-4* the 4-item patient health questionnaire, *FRAS* Family Resilience Assessment Scale

### Correlation analysis

Additional file 1 presents the correlations among caregivers’ variables, with all associations statistically significant. Caregiver burden, psychological distress and its sub-dimensions exhibited negative correlations with family resilience (*P* < 0.01) and QoL (*P* < 0.01). Conversely, family resilience (FR) demonstrated a positive correlation with QoL (*P* < 0.01).

### Hypotheses testing

#### The mediation models

Hypothesis 1 posited the mediation effect of psychological distress on the association between caregiver burden and QoL. The results are presented in Table 3, which displays the total, direct and indirect effects of the relationship between caregiver burden and QoL through psychological distress and its sub-dimensions. The analysis was adjusted for caregivers’ sex and the presence of chronic diseases (found in the regression model of QoL, see Additional file 2). Controlling for psychological distress, the relationship between caregiver burden and QoL remained statistically significant (direct effect) (β = -0.352, *P* < 0.001). Additionally, the effect of psychological distress in explaining the correlation between caregiver burden and QoL was also statistically significant (indirect effect) (β = -0.204, *P* < 0.001), supporting evidence of partial mediation. Similarly, when the mediator was depression or anxiety, both the direct (β = -0.386, -0.387, respectively, *P* < 0.001) and indirect effects (β = -0.169, -0.170, respectively, *P* < 0.001) were statistically significant, further indicating partial mediation.

**Table 3:** Total, direct and indirect effects of mediating role of psychological distress

Path	Total effect	Direct effect	Indirect effect
CGB → PD → QoL	-0.556 [-0.661, -0.452]	-0.352 [-0.452, -0.253]	-0.204 [-0.292, -0.128]
CGB → Dep → QoL	-0.556 [-0.661, -0.452]	-0.386 [-0.488, -0.286]	-0.169 [-0.245, -0.106]
CGB → Anx → QoL	-0.556 [-0.661, -0.452]	-0.387 [-0.486, -0.287]	-0.170 [-0.253, -0.098]

Control variables: caregivers’ sex and the presence of chronic diseases

*CGB* Caregiver burden, *PD* Psychological distress, *QoL* QoL, *Dep* Depression, *Anx* Anxiety

#### The moderation models

**Fig. 2:**
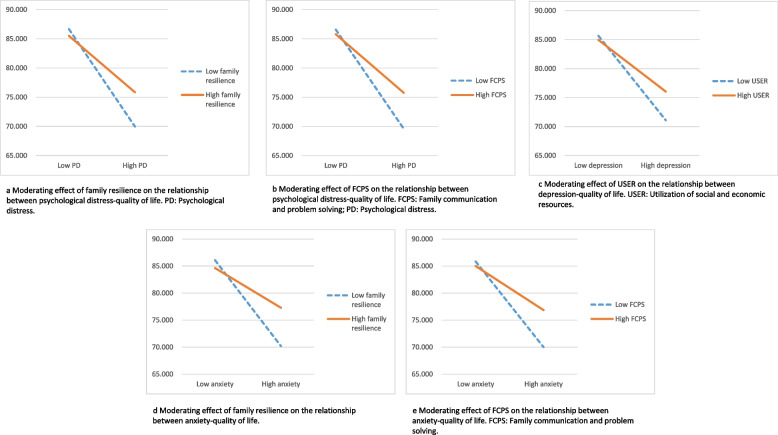
**a** Moderating effect of family resilience on the relationship between psychological distress-quality of life. PD: Psychological distress. **b** Moderating effect of FCPS on the relationship between psychological distress-quality of life. FCPS: Family communication and problem solving; PD: Psychological distress. **c** Moderating effect of USER on the relationship between depression-quality of life. USER: Utilization of social and economic resources. **d** Moderating effect of family resilience on the relationship between anxiety-quality of life. **e** Moderating effect of FCPS on the relationship between anxiety-quality of life. FCPS: Family communication and problem solving

Hypothesis 2, which examined the moderating effect of family resilience and its sub-dimensions on the mediation models of “caregiver burden-psychological distress/depression/anxiety-QoL”, only found statistically significant moderating effects in the second paths. The significant results were displayed in Table 4. Figure 2 showed that the impact of psychological distress, depression, or anxiety on QoL varied according to the level of family resilience and its sub-dimensions among caregivers. Figure 2a showed that the interaction term for psychological distress with family resilience (PD*FR) was statistically significant in predicting caregivers’ QoL, Fig. 2b demonstrated the statistical significance of the interaction term for psychological distress with FCPS (PD*FCPS) in predicting QoL. Additionally, Fig. 2c displayed the significant interaction term for depression with USER (Dep*USER) in predicting QoL. Moreover, both Fig. 2d and e indicated that the interaction terms for anxiety with family resilience (Anx*FR) and anxiety with FCPS (Anx*FCPS) were statistically significant in predicting caregivers’ QoL. These findings indicated that when family resilience were at lower levels, the negative impact of psychological distress, depression, or anxiety on QoL was stronger. In comparison, at higher levels of family resilience, the adverse effects of psychological distress, depression, or anxiety on QoL weakened, providing evidence of the moderating effect of family resilience.

**Table 4:** The moderating effect of family resilience on the mediating pathway of "caregiving burden-psychological distress-QoL"

	*R* ^2^	*F*	*β*	*SE*	*t*	*P*	LLCI	ULCI
*Caregiver burden-Psychological distress-QoL (Model 14) Outcome: QoL*	0.497	46.669^***^						
Caregiver burden (CGB)			-0.351	0.050	-6.977	< 0.001	-0.450	-0.252
Psychological distress (PD)			-2.506	0.286	-8.770	< 0.001	-3.069	-1.944
Family resilience (FR)			0.084	0.050	1.682	0.094	-0.014	0.183
PD*FR			0.048	0.020	2.374	0.018	0.008	0.088
*Caregiver burden-Psychological distress-QoL (Model 14) Outcome: QoL*	0.498	46.760^***^						
Caregiver burden (CGB)			-0.349	0.050	-6.939	< 0.001	-0.447	-0.250
Psychological distress (PD)			-2.573	0.279	-9.220	< 0.001	-3.122	-2.023
Family communication and problem solving (FCPS)			0.168	0.087	1.926	0.055	-0.004	0.340
PD*FCPS			0.083	0.035	2.340	0.020	0.013	0.152
*Caregiver burden-Depression-QoL (Model 14) Outcome: QoL*	0.462	40.482^***^						
Caregiver burden (CGB)			-0.391	0.051	-7.627	< 0.001	-0.492	-0.290
Depression (Dep)			-4.338	0.564	-7.687	< 0.001	-5.449	-3.227
Utilization of social and economic resources (USER)			0.362	0.240	1.511	0.132	-0.110	0.834
Dep*USER			0.358	0.175	2.049	0.041	0.014	0.702
*Caregiver burden-Anxiety-QoL (Model 14) Outcome: QoL*	0.477	43.085^***^						
Caregiver burden (CGB)			-0.384	0.051	-7.597	< 0.001	-0.484	-0.285
Anxiety (Anx)			-3.926	0.508	-7.725	< 0.001	-4.926	-2.925
Family resilience (FR)			0.101	0.051	1.994	0.047	0.001	0.201
Anx*FR			0.104	0.038	2.715	0.007	0.029	0.179
*Caregiver burden-Anxiety-QoL (Model 14) Outcome: QoL*	0.475	42.723^***^						
Caregiver burden (CGB)			-0.383	0.051	-7.562	< 0.001	-0.482	-0.283
Anxiety (Anx)			-4.056	0.500	-8.118	< 0.001	-5.039	-3.072
Family communication and problem solving (FCPS)			0.187	0.089	2.108	0.036	0.012	0.362
Anx*FCPS			0.162	0.067	2.432	0.016	0.031	0.294

Control variables: caregiver sex, the presence of chronic disease. The model was adjusted by caregivers’ sex and the presence of chronic diseases

*SE* Standard error, *LLCI* Lower limit of 95% confidence interval, *ULCI* Upper limit of 95% confidence interval, *CGB* Caregiver burden, *PD* Psychological distress, *FR* Family resilience, *Dep* Depression, *Anx* Anxiety, *FCPS* Family communication and problem solving, *USER* Utilization of social and economic resources

^***^ *P* < 0.001

Additionally, a test of the overall model was conducted by examining the conditional indirect effect (moderated mediation) of psychological distress, depression, or anxiety on the relationship between caregiver burden and QoL at varying levels of family resilience or its sub-dimensions. The results are presented in Table 5, which displays the estimates, standard errors, and bootstrap confidence intervals for the conditional indirect effects of caregiver burden across low and high levels of family resilience.

**Table 5:** Moderated mediation results of caregiver burden for varying levels of family resilience or its sub-dimensions

Moderator	Conditional effect	SE	LLCI	ULCI
*FR (Mediator: PD)*				
Low (-1SD)^a^	-0.238	0.051	-0.345	-0.145
Mean^b^	-0.188	0.038	-0.271	-0.119
High (+ 1SD)^c^	-0.138	0.039	-0.222	-0.068
*FCPS (Mediator: PD)*				
Low (-1SD)^a^	-0.243	0.052	-0.349	-0.147
Mean^b^	-0.193	0.039	-0.273	-0.122
High (+ 1SD)^c^	-0.143	0.038	-0.221	-0.072
*USER (Mediator: Depression)*				
Low (-1SD)^a^	-0.195	0.044	-0.285	-0.116
Mean^b^	-0.157	0.034	-0.230	-0.096
High (+ 1SD)^c^	-0.119	0.035	-0.194	-0.056
*FR (Mediator: Anxiety)*				
Low (-1SD)^a^	-0.208	0.050	-0.311	-0.117
Mean^b^	-0.152	0.036	-0.229	-0.088
High (+ 1SD)^c^	-0.096	0.040	-0.185	-0.029
*FCPS (Mediator: Anxiety)*				
Low (-1SD)^a^	-0.208	0.051	-0.314	-0.116
Mean^b^	-0.157	0.037	-0.236	-0.091
High (+ 1SD)^c^	-0.107	0.041	-0.195	-0.036

*FR* Family resilience, *PD* Psychological distress, *SD* Standard deviation, *SE* Standard error, *LLCI* Lower limit of 95% confidence interval, *ULCI* Upper limit of 95% confidence interval, *FCPS* Family communication and problem solving, *USER* Utilization of social and economic resources

^a^The value of the moderating variable (FR, FCPS, or USER) is one standard deviation below the mean

^b^The value of the moderating variable (FR, FCPS, or USER) is at the mean

^c^The value of the moderating variable (FR, FCPS, or USER) is one standard deviation above the mean

## Discussion

This study illuminates the intricate relationship between caregiver burden, psychological distress and its sub-dimensions of anxiety and depression, family resilience, and the QoL among caregivers of advanced cancer patients. Previous research primarily concentrated on the direct relationship between caregiver burden and QoL [8], neglecting to explore the underlying mechanisms at play. To fill this knowledge gap, this study proposed and evaluated the mediating role of psychological distress, including anxiety and depression, in the connection between caregiver burden and QoL. Further, guided by the cognitive appraisal theory of stress [18] and the family strengths perspective [13, 19], the moderating role of family resilience was probed. The findings substantiate both hypotheses, demonstrating a significant indirect path from caregiver burden via psychological distress to QoL, thereby confirming the mediation effect of psychological variables. Additionally, significant interaction effects between psychological distress (and its sub-dimensions) and caregivers' family resilience (and its sub-dimensions) on QoL were identified. Importantly, the intensity of the adverse effects of psychological distress on QoL fluctuated at varying levels of family resilience, particularly within the sub-dimensions of FCPS, and USER. These insights suggest that interventions striving to augment caregivers' QoL should consider addressing both psychological factors and promoting family resilience.

This research reveals that caregivers of advanced cancer patients typically manifest a moderate to above-average overall QoL. However, caregivers burdened with more intensive caregiving responsibilities endure markedly elevated levels of psychological distress, encompassing increased severity of depression and anxiety. This relationship corroborates previous research, highlighting that caregiver burden escalates in tandem with psychological distress, thereby affecting the caregivers' psychological well-being [4, 27]. Health care providers should, therefore, develop tailored interventions that provide mental health support and alleviate caregiving burden, enabling caregivers to navigate effectively through caregiving challenges.

Consistent with prior studies [5, 28], the findings of this study establish a significant negative association between psychological distress and caregivers' QoL. Evidence shows that psychological interventions addressing caregiver anxiety or depression effectively enhance their overall QoL [29]. Caregivers under significant psychological distress may perceive elevated stress levels and a reduced sense of control, substantially impacting their QoL [30]. The current research further supports the mediator role of psychological distress between caregiving burden and QoL. Caregiving burden positively predicts psychological distress, which then adversely impacts caregivers' QoL. Such pattern is pronounced among caregivers of advanced cancer patients, likely due to the disease's complexity and demanding treatment [31]. Overwhelmed by caregiving tasks, caregivers may experience an intense personal and emotional burden, leading to anxiety and/or depression, further deteriorating their QoL [32, 33]. These findings underscore the importance of developing interventions targeting psychological distress to mitigate the adverse impact of caregiving burden on caregivers' QoL. Such interventions, encompassing psychological support and appropriate training, can equip caregivers to handle their caregiving tasks and psychological distress more efficiently, thereby enhancing their QoL [34]. In designing personalized care plans, healthcare providers should consider the mental health of caregivers and provide them with the necessary support and resources to ensure their well-being while fulfilling their caregiving duties.

This study supports the premise that family resilience moderates the relationship between psychological distress and QoL. This aspect, relatively under-researched in prior studies, is addressed in this investigation, thereby filling an important gap in the literature. Specifically, heightened levels of family resilience were observed to attenuate the adverse impact of psychological distress or anxiety on QoL. This observation may be attributed to the family's capacity to confront and recover from adversity, as encapsulated in their resilience [11–13]. Caregivers perceiving higher levels of family resilience suggest their families are better equipped to manage the challenges posed by advanced cancer [35]. Consequently, they are more adept at navigating the impact of advanced cancer on their family and its members. This proficiency mitigates caregivers' levels of psychological distress or anxiety, resulting in an enhancement in their overall QoL. Importantly, it must be stressed that advanced cancer influences the entire family [36], thus necessitating the consideration of family-level factors, specifically family resilience, during psychological interventions for caregivers of advanced cancer patients. The integration of family into caregiving interventions is paramount, as family support and coping mechanisms are intimately linked to caregivers' health outcomes. Healthcare professionals should prioritize the assessment of psychological distress, especially anxiety levels among family caregivers while simultaneously evaluating their family resilience levels. Implementing strategies to fortify family resilience, such as providing family support and education and enhancing intra-family communication and interaction, can empower caregivers to better confront their challenges, ultimately improving their overall QoL.

The exploration of the moderating effects of various dimensions of family resilience revealed that FCPS, an essential facet of family resilience, can buffer the negative impact of psychological distress or anxiety on caregivers' QoL. These findings underscore the pivotal role of family resilience in promoting caregivers' psychological well-being and overall QoL. In the context of advanced cancer caregiving, effective family communication facilitates the exchange and comprehension of information [37, 38], thereby reducing the psychological distress stemming from inadequate caregiving skills and uncertainties regarding the patient's illness [39, 40]. Encouraging open communication allows caregivers to express their emotions and needs and understand the patient's condition and requirements, leading to a reduction in psychological distress [41–43]. Moreover, families coping with advanced cancer frequently encounter numerous challenges and dilemmas, such as the division of caregiving responsibilities and making medical decisions [44–46]. A proficiency in problem-solving enables family members to collaboratively address these challenges and devise optimal solutions [47, 48]. Consequently, caregivers' psychological distress, especially anxiety levels, is alleviated, enhancing their confidence in caregiving and ultimately improving their overall QoL. As healthcare providers, the responsibility lies in encouraging and facilitating open communication among caregivers and family members, equipping them with effective communication skills and problem-solving strategies [49]. Through targeted education and support, caregivers are expected to be better prepared to handle challenges, reduce psychological distress, and elevate their QoL.

Furthermore, the findings of this study reveal that the utilization of social and economic resources can mitigate the adverse impact of caregiver depression on their QoL. This component of family resilience revolves around the effective leveraging of social and economic resources by family members to combat stress and adversity [13]. Caregivers of patients with advanced cancer may encounter economic burdens, time constraints, and resource limitations, which can augment their psychological stress [50, 51]. Consequently, when caregivers suffer from depression, the family's burden may be intensified due to sustained stress. Nevertheless, this dimension of family resilience underscores the necessity of optimizing the use of social and economic resources [13]. Such resources include engaging professional nursing services, participating in support groups, and securing financial assistance [52, 53]. These measures can accommodate caregivers' needs, reduce psychological distress, and thereby enhance their overall QoL. Healthcare providers play an integral role in offering information and guidance regarding social and economic resources throughout the caregiving process for patients with advanced cancer [54, 55]. Assisting caregivers in comprehending how to effectively utilize social resources, including seeking community support and participating in support organizations, is indispensable. Moreover, the nursing team can offer valuable support to caregivers in securing financial aid, which would facilitate improved coping with caregiving responsibilities and ultimately enhance their QoL.

### Implications

This study suggests that caregiver burden among caregivers of advanced cancer patients negatively impacts their QoL, predominantly via psychological distress, including its sub-dimensions, namely anxiety and depression. However, family resilience, as well as its sub-dimensions, especially FCPS and USER, plays a significant role in mitigating the impact of psychological distress on their QoL. These findings indicate that by implementing personalized intervention strategies, providing psychological support, and fostering family resilience, healthcare professionals can strengthen caregivers' coping mechanisms and enhance their QoL. Specifically, nurturing open communication and problem-solving skills among caregivers and family members emerges as a crucial strategy, particularly beneficial for individuals experiencing psychological distress or anxiety. Simultaneously, facilitating access to social and economic resources can lead to positive outcomes, especially for those grappling with depression. Providing comprehensive support and care to family members throughout the caregiving journey can enrich caregivers' experiences and elevate the quality of care, ultimately fostering the well-being and happiness of the entire family.

### Strengths and limitations

This study serves to affirm the mediating role of psychological distress in the relationship between caregiver burden and QoL, whilst simultaneously highlighting the vital role of family resilience as a moderator. These findings enrich the domain of family resilience studies and provide useful insights for family-focused nursing interventions. However, the study has certain limitations. Firstly, being a cross-sectional study, it cannot establish causal relationships. Secondly, it is possible that the caregivers who participated in the study were more proactive, potentially leading to an overestimation of family resilience's role and introducing response bias. Furthermore, family resilience may fluctuate over time, with different aspects becoming prominent at various stages, an aspect this study did not consider. Future longitudinal studies might explore the moderating effects of family resilience on caregiver burden and QoL at different stages. Additionally, this study was centered solely on caregivers, neglecting the influence of other patient-related variables, dyadic factors, and social factors such as dyadic coping and social support [56, 57]. To gain a more nuanced understanding of the targets for improving caregiver QoL, future research should consider these factors, thereby providing a foundation for designing intervention studies.

## Conclusions

This study examined the relationship between caregiver burden and QoL among caregivers of advanced cancer patients, employing moderated mediation models. There was a significant negative correlation between caregiver burden and QoL, with psychological distress and its sub-dimensions— specifically anxiety and depression—mediating the relationship between caregiver burden and QoL. Both the psychological distress-mediated model and the anxiety-mediated model were moderated by family resilience as well as its sub-dimension—FCPS. Additionally, the depression-mediated model was moderated by USER, another specific sub-dimension of family resilience. These findings underscore the importance of considering not only the direct impact of caregiver burden on QoL but also the intricate interplay involving psychological distress and family resilience. Future research should consider patient-related variables, dyadic factors, and social factors to fully explore potential avenues for improving caregiver QoL and to establish a foundation for effective intervention strategies.

### Supplementary Information

**Supplementary Material 1.**

**Supplementary Material 2.**

## Data Availability

Data and analytical methods in this study are available from the corresponding author upon reasonable request.

## Abbreviations

AMMA: Ability to make meaning of adversity
Anx: Anxiety
CGB: Caregiver burden
CI: Confidence interval
Dep: Depression
FC: Family connectedness
FCPS: Family communication and problem solving
FR: Family resilience
FRAS: Family Resilience Assessment Scale
FS: Family spirituality
MCS: Mental component summary
MPO: Maintaining a positive outlook
PCS: Physical component summary
PD: Psychological distress
PHQ-4: The 4-item patient health questionnaire
QoL: Quality of life
SD: Standard deviation
SF-8: The short form 8 health survey
USER: Utilization of social and economic resources
ZBI: Zarit Burden Interview
