# Supplementary material for: The impact of caregiver burden on quality of life in family caregivers of patients with advanced cancer: a moderated mediation analysis of the role of psychological distress and family resilience
Source: BMC Public Health. 2024 Mar 15;24:817. doi: 10.1186/s12889-024-18321-3 (PMC10941369; doi:10.1186/s12889-024-18321-3)
Supplement: Supplementary file 1 — Supplementary Material 1. [file 12889_2024_18321_MOESM1_ESM.docx]

Additional file 1 Correlations among variables (*N*=290)

| Variables | QoL | CGB | PD | Dep | Anx | FR |
| --- | --- | --- | --- | --- | --- | --- |
| QoL | 1 |  |  |  |  |  |
| CGB | -0.538** | 1 |  |  |  |  |
| PD | -0.603** | 0.418** | 1 |  |  |  |
| Dep | -0.554** | 0.393** | 0.921** | 1 |  |  |
| Anx | -0.564** | 0.383** | 0.934** | 0.708** | 1 |  |
| FR | 0.228** | -0.211** | -0.242** | -0.226** | -0.229** | 1 |
| QoL: Quality of life; CGB: Caregiver burden; PD: Psychological distress; Dep: Depression; Anx: Anxiety; FR: Family resilience. | | | | | | |
