# Supplementary material for: The impact of caregiver burden on quality of life in family caregivers of patients with advanced cancer: a moderated mediation analysis of the role of psychological distress and family resilience
Source: BMC Public Health. 2024 Mar 15;24:817. doi: 10.1186/s12889-024-18321-3 (PMC10941369; doi:10.1186/s12889-024-18321-3)
Supplement: Supplementary file 2 — Supplementary Material 2. [file 12889_2024_18321_MOESM2_ESM.docx]

Additional file 2 Regression analysis of caregiver quality of life (n=290)

|  | *β* | *SE* | Std. *β* | *t* | *P* | LLCI | ULCI |
| --- | --- | --- | --- | --- | --- | --- | --- |
| Constant | 89.540 | 10.625 |  | 8.427 | 0.000 | 68.621 | 110.458 |
| Sex (patient) | 2.079 | 1.423 | 0.068 | 1.461 | 0.145 | -0.723 | 4.881 |
| Age (patient) | -1.406 | 1.221 | -0.072 | -1.152 | 0.250 | -3.810 | 0.997 |
| Primary cancer (patient) | 0.717 | 1.635 | 0.020 | 0.439 | 0.661 | -2.502 | 3.936 |
| Time since advanced cancer diagnosis/month (patient) | 0.890 | 1.445 | 0.029 | 0.616 | 0.538 | -1.954 | 3.735 |
| Sex | 3.697 | 1.393 | 0.121 | 2.654 | **0.008** | 0.954 | 6.439 |
| Age | 0.467 | 1.251 | 0.022 | 0.373 | 0.709 | -1.995 | 2.929 |
| Marital status | 1.316 | 2.242 | 0.028 | 0.587 | 0.558 | -3.099 | 5.731 |
| Education level | 1.001 | 0.929 | 0.054 | 1.078 | 0.282 | -0.827 | 2.830 |
| Working status | -2.498 | 1.597 | -0.081 | -1.564 | 0.119 | -5.641 | 0.646 |
| Average household income per capita (RMB) | 0.086 | 0.828 | 0.005 | 0.104 | 0.917 | -1.544 | 1.716 |
| The presence of chronic diseases | -4.210 | 1.853 | -0.106 | -2.272 | **0.024** | -7.859 | -0.561 |
| Whether they had similar caregiving experience | -2.472 | 1.613 | -0.068 | -1.533 | 0.126 | -5.648 | 0.703 |
| Type of caregiving | -1.853 | 1.516 | -0.060 | -1.223 | 0.223 | -4.838 | 1.131 |
| Length of care (month) | 0.065 | 0.920 | 0.004 | 0.071 | 0.943 | -1.747 | 1.878 |
| Caregiving hours per day | 0.526 | 0.582 | 0.041 | 0.904 | 0.367 | -0.620 | 1.672 |
| Relationship patient-caregiver (spouse) | 0.598 | 2.179 | 0.019 | 0.274 | 0.784 | -3.693 | 4.888 |
| Relationship patient-caregiver (adult children) | 0.320 | 2.277 | 0.010 | 0.141 | 0.888 | -4.162 | 4.803 |
| Caregiver burden | -0.335 | 0.052 | -0.314 | -6.470 | 0.000 | -0.437 | -0.233 |
| Psychological distress | -2.756 | 0.291 | -0.473 | -9.480 | 0.000 | -3.329 | -2.184 |
| Family resilience | 0.059 | 0.050 | 0.053 | 1.174 | 0.242 | -0.040 | 0.157 |
| R2=0.526, adjusted R2=0.491, *F*=14.950, *P*<0.001. Std. *β*=Standardized β; LLCI: Lower limit of 95% confidence interval; ULCI: Upper limit of 95% confidence interval. | | | | | | | |
